# BCL10GFP fusion protein as a substrate for analysis of determinants required for Mucosa-Associated Lymphoid Tissue 1 (MALT1)-mediated cleavage

**DOI:** 10.1186/1423-0127-19-85

**Published:** 2012-10-05

**Authors:** Shin-Yi Jou, Chien-Chih Chang, Chun-Hsien Wu, Mei-Ru Chen, Ching-Hwa Tsai, Wen-Hui Chuang, Yun-Hui Chen, Ann-Lii Cheng, Shin-Lian Doong

**Affiliations:** 1 Graduate Institute of Microbiology, College of Medicine, National Taiwan University, No.1, Section 1, Jen-Ai Road, Taipei 10051, Taiwan; 2 Department of Oncology and Internal Medicine, National Taiwan University Hospital, No.7, Chung San South Road, Taipei, Taiwan; 3 Cancer Research Center, College of Medicine, National Taiwan University, Taipei, Taiwan

**Keywords:** Paracaspase, MALT1, BCL10GFP, *In vivo*, Processing

## Abstract

**Background:**

MALT1 belongs to a family of paracaspase and modulates NF-κB signaling pathways through its scaffolding function and proteolytic activity. MALT1 cleaves protein substrates after a positively charged Arginine residue. BCL10, a 233 amino acids polypeptide, is identified as one of the MALT1 proteolytic substrates. MALT1 cleaves BCL10 at the C-terminal end of Arg228. A mere 5 amino acids difference between the substrate and the proteolytic product made it difficult to tell whether the cleavage event took place by using a simple western blot analysis. Here, BCL10GFP was constructed and utilized to examine the specificity and domain determinants for MALT1 cleavage in cells.

**Methods:**

Various BCL10GFP constructs were transfected into HEK293T cell with MALT1 construct by using calcium phosphate-DNA precipitation method. Lysates of transfectants were resolved by SDS/PAGE and analyzed by western blot analysis.

**Results:**

BCL10GFP was proteolytically processed by MALT1 as BCL10. The integrity of caspase recruitment domain (CARD) and MALT1-interacting domain on BCL10 were required for MALT1 proteolytic activity. Besides the invariant P1 cleavage site Arg228, P4 Leu225 played a role in defining BCL10 as a good substrate for MALT1.

**Conclusions:**

We offered a way of monitoring the catalytic activity of MALT1 in HEK293T cells using BCL10GFP as a substrate. BCL10GFP can be utilized as a convenient tool for studying the determinants for efficient MALT1 cleavage in HEK293T cells

## Background

Human MALT1 (Mucosa-associated lymphoid tissue 1) contains 824 amino acid residues and an N-terminal death domain (DD), two immunoglobulin-like (Ig-like) domains, followed by a C-terminal caspase-like-domain (CLD) [1,2] and a third Ig-like domain [3]. MALT1, together with BCL10, has been shown to couple with different CARMA (CARD containing MAGUK protein) family protein to assemble into the CBM complex to transduce NF-κB signaling downstream of multiple receptors [4-8]. MALT1 links the CBM complex to IKK activation by the recruitment of TRAF6 [3,9]. Besides its first-identified scaffolding function, the catalytic activity of MALT1 and the biological consequences resulting from its proteolytic activation have been topics of great interest. It has long been known that MALT1 constructs with mutations in the predicted active site showed a significant reduction in their capacity to activate NF-κB [2,10]. BCL10 was found to be a proteolytic substrate of MALT1 [11]. However, proteolytic processing of BCL10 is associated with the fibronectin adhesion and not required for NF-κB activation [11]. It was not until 2008 that a mechanistic connection between MALT1 protease activity and NF-κB activation was demonstrated by Coornaert et al. [12]. They provided evidences that NFκB signaling was enhanced from A20 cleavage by MALT1 [12]. Lately, CYLD [13] and RelB [14] were also reported to be proteolytic targets of MALT1. In addition, NIK was shown to be processed by IAP2-MALT1 fusion oncoprotein [15].

By examining the MALT1 cleavage sites in these substrates, they share a common P2-P1 preference for Ser-Arg. Unlike caspases, which cleave specific substrate sequences immediately after Asp residues, MALT1 paracaspase recognizes a substrate sequence with Arg immediately N-terminal to the cleavage site. Lately, the structure of human MALT1 paracaspase bonded with a peptide inhibitor was published [16,17]. Both studies suggested for Arg invariant at the P1 position and preference for a hydrophobic residue at P4 position. In this study, BCL10 was fused at the C-terminus with green fluorescence protein, generating BCL10GFP fusion protein, to study the determinants required for MALT1 cleavage in HEK293T cells.

## Methods

### Plasmids and antibodies

EST clone IMAGE:703916, containing the ORF of *BCL10* gene was purchased form Invitrogen (Carlsbad, CA) and utilized as template for construction of all the BCL10-derived expression vectors. Expression vectors **pCMV6/XL5/MALT1** (containing full length MALT1 cDNA) and **pCMV6/XL5/IAP2** (Containing full length IAP2 cDNA) were purchased from ORIGENE Technologies Inc. (Rockville, Maryland). Please see the supplementary for detailed construction information for all BCL10, IAP2-MALT1 and MALT1 constructs. All the constructs were confirmed by sequencing analysis.

Mouse anti-BCL10 monoclonal antibody (sc-5273) and mouse anti-MALT1 antibody (sc-46677) were purchased from Santa Cruz Biotchnology (Santa Cruz, CA). Anti-GAPDH antibody (H86504M) was purchased from Biodesign international (Saco, ME). Rabbit polyclonal antibody against GFP expressed in *E. coli* was also generated for detection of green fluorescence protein. Horse radish peroxidase-linked donkey anti-rabbit IgG and sheep anti-mouse IgG were purchased from Amersham Biosciences.

### Cell Culture and Transfection

HEK293T cells were cultured in DMEM containing 10% (vol/vol) FBS, 50 units/ml penicillin, 50 μg/ml streptomycin, 1.25 μg/ml fungizone (all from GIBCO, Invitrogen, Carlsbad, CA) at 37°C in a 5% CO_2_ incubator. Transfection was performed by the calcium phosphate-DNA precipitation method.

### Western Blot Analysis

Cells were rinsed with PBS and lysed with RIPA buffer (150 mM NaCl, 50 mM Tris pH7.5,1% NP40, 0.1% SDS, 1mM EDTA, 1mM PMSF, 1μg/ml aprotinin, leupeptin, pepstatin, 1mM Na_3_VO_4_, 1mM NaF). Denatured protein were separated on SDS/polyacrylamide gels, transferred to Hybond-P membranes (Amersham Biosciences), and subjected to immunoblotting with antibodies indicated.

### Immunoprecipitation and CIAP Treatment

Cells were rinsed with PBS and lysed with modified RIPA buffer (150 mM NaCl, 50 mM Tris pH7.5, 1% NP40, 0.25% Sodium deoxycholate, 1mM EDTA, 1mM PMSF, 1mg/ml aprotinin, leupeptin, pepstatin, 1mM Na_3_VO_4_, 1mM NaF). 1 mg total cellular protein were incubated with 5 μl mouse anti-BCL10 antibody (sc-5273, Santa Crutz Biotchnology) for 16 hr at 4°C. 50μl protein A beads were then added. The whole mixtures were incubated at 4°C for 4 hr. Protein A beads were spun down, washed twice with RIPA buffer and subjected to Calf Intestine Alkaline Phosphatase (CIAP) (New England Biolab.) treatment as suggested by the manufacturer’s protocol.

### Protein expression and purification

pET21aBCL10-His and pET21aBCL10-His mutants were transformed into BL21(DE3) *E. coli* cells. Protein expression was induced with 1 mM IPTG (isopropyl β-D-thiogalactopyranoside) for 4 hr at 37°C. *E. coli* cells were lysed in lysis buffer (50 mM NaH_2_PO_4_ pH 8.0, 300 mM NaCl, 10 mM imidazole, 8 M urea), sonicated. The lysates were centrifuged at 13K rpm (KUBOTA 1920) for 10 min at 4°C. The soluble fraction was applied to Ni^2+^ NTA agarose (Qiagen). Purification was performed as the manufacture’s instruction. The protein was eluted with 250 mM imidazole.

pET21a-MALT1-His or pET21a-MALT1C464A-His was transformed into Arctic-Express^TM^ RIL compent *E. coli* cells. Protein expression was induced with 1 mM IPTG (isopropyl β-D-thiogalactopyranoside) for 48 hr at 8°C. *E. coli* cells were suspended in buffer (50 mM NaH_2_PO_4_ pH 8.0, 300 mM NaCl, 10 mM imidazole) and lysed by 700 psi french press (Thermo IEC FRENCH press laboratory with mini pressure cell, 120VAC, 60Hz). The lysates were centrifuged at 13K rpm (KUBOTA 1920) for 10 min at 4°C. The soluble fraction was applied to Ni^2+^ NTA agarose (Qiagen). The protein was purified according to the manufacture’s instruction and eluted with 250 mM imidazole.

All the purified proteins were dialyzed against PBS and stored at -70°C freezer in the presence of 20% glycerol.

### *In vitro* cleavage assay of purified MALT1

50 ng purified BCL10 or mutant BCL10 proteins were incubated with 1 μg or 2 μg purified full length MALT1 or catalytic-inactive mutant MALT1C464A respectively for 4 hr at 30°C in 50 mM MES (pH 6.8), 150 mM NaCl, 10% sucrose, 0.1% CHAPS, 10 mM DTT, and 1 M ammonium citrate. The reaction was subsequently analyzed by SDS/PAGE, followed by Western blotting with an anti-BCL10 antibody or with an anti-MALT1 antibody. Blots were scanned using UVP Biospectrum AC imaging system. The percentage of BCL10 processed was defined as the signal of cleaved product divided by the sum of signal of full-length protein and the cleaved product.

## Results

### BCL10GFP was proteolytically processed by MALT1 as BCL10

**Figure 1:**
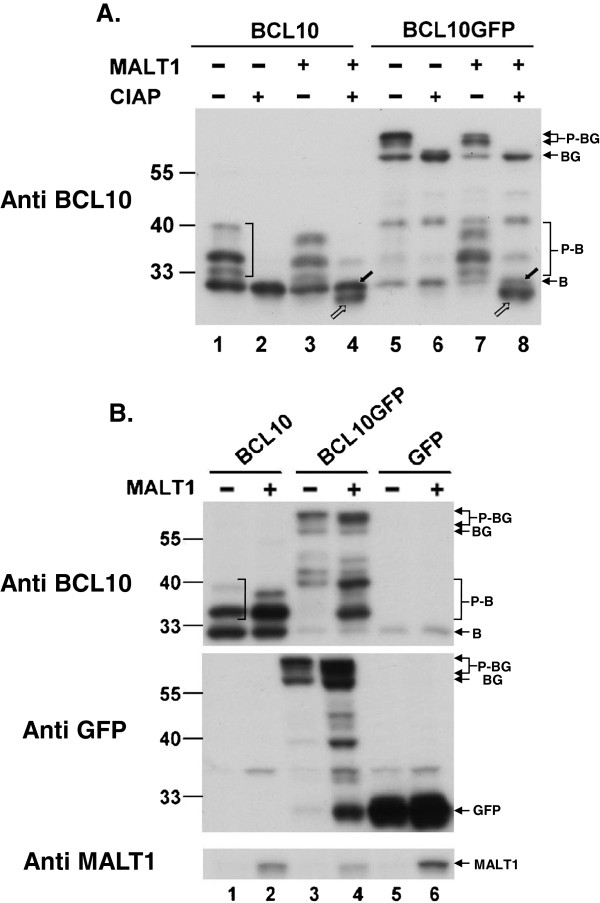
**Proteolytic cleavage of BCL10GFP by MALT1.** (**A**) Lysates of HEK293T cells transfected with BCL10 or BCL10GFP in the absence (-) or the presence (+) of MALT1 as indicated were immunoprecipitated using anti-BCL10 antibody and treated with or without calf intestine alkaline phosphatase (CIAP), and analyzed by Western blotting with anti-BCL10 antibody. filled arrow: full length BCL10; unfilled arrow: truncated BCL10 (**B**) 2×10^5^ HEK293T cells were transfected with 0.25 μg of pRc/CMVBCL10, pRc/CMVBCL10GFP, or pRc/CMVGFP and 2 μg of pCMV6/XL5 or pCMV6/XL5/MALT1. Western blot analysis of total cell lysates was carried out using anti-BCL10, anti-GFP and anti-MALT1antibodies. B: BCL10; P-B: phosporylated BCL10; BG: BCL10GFP; P-BG: Phosphorylated BCL10GFP.

BCL10 was phosphorylated intracellularly and presented as multiple slowly-migrating forms in the SDS/polyacrylamide gel (Figure 1A, lane 1; Figure 1B, lane 1). MALT1-induced cleavage products were difficult to be detected as the migration pattern of BCL10 from cells with co-expression of MALT1 was not obviously changed (Figure 1A, lane 3; Figure 1B, lane 2). Phosphatase treatment converted multiple forms to a single unmodified BCL10 (Figure 1A, lane 2) from lysates of cells with BCL10 overexpression. Whereas in cells co-transfected with MALT1, phosphatase treatment revealed the apparent emergence of truncated BCL10 under the unmodified BCL10 (Figure 1A, lane 4). Without phosphatase treatment, the MALT1-mediated cleavage event on BCL10 could not be easily identified using a simple western blot analysis.

MALT1-mediated cleavage occurs at a single site 5 amino acid from the carboxy-terminal end of BCL10 [11]. By fusion of 25 kdalton green fluorescence protein (GFP) at the carboxy end of BCL10, the cleavage event could be easily monitored by the appearance of truncated BCL10 and a form with 5 amino acids from BCL10 fused at the N-terminus of GFP. BCL10GFP, like BCL10, was hyperphosphorylated intracellularly and presented as multiple forms near 55 kdalton (Figure 1A, lane 5; Figure 1B, lane 3). In addition to these hyperphosphorylated forms of BCL10GFP, multiple forms migrating between 33 and 40 kdalton were consistently observed in lysates from cells expressing both BCL10GFP and MALT1 (Figure 1A, lane 7; Figure 1B, lane 4). As shown in Figure 1A (lanes 7, 8), phosphatase treatment converted MALT1-induced forms to a form as the truncated BCL10 migrating below the endogenous non-modified BCL10. A form with molecular weights near 25 kdalton, where GFP was expected to appear (Figure 1B,middle panel, lanes 5-6), was also detected using anti GFP antibodies (Figure 1B, middle panel, lane 4) in lysates from cells cotransfected with BCL10GFP and MALT1 but not from cells with expression of BCL10GFP only (Figure 1B,middle panel, lane 3). These results indicated that BCL10GFP could be proteolytically processed by MALT1 as BCL10. The appearance of a form near 25 kdalton using anti-GFP antibody and multiple forms between 33 and 40 kdalton using anti-BCL10 antibody could be utilized as indicators for the occurrence of a MALT1-mediated cleavage event.

### Both the Ig-like and caspase-like-domains of MALT1 were required for mediating BCL10GFP cleavage

**Figure 2:**
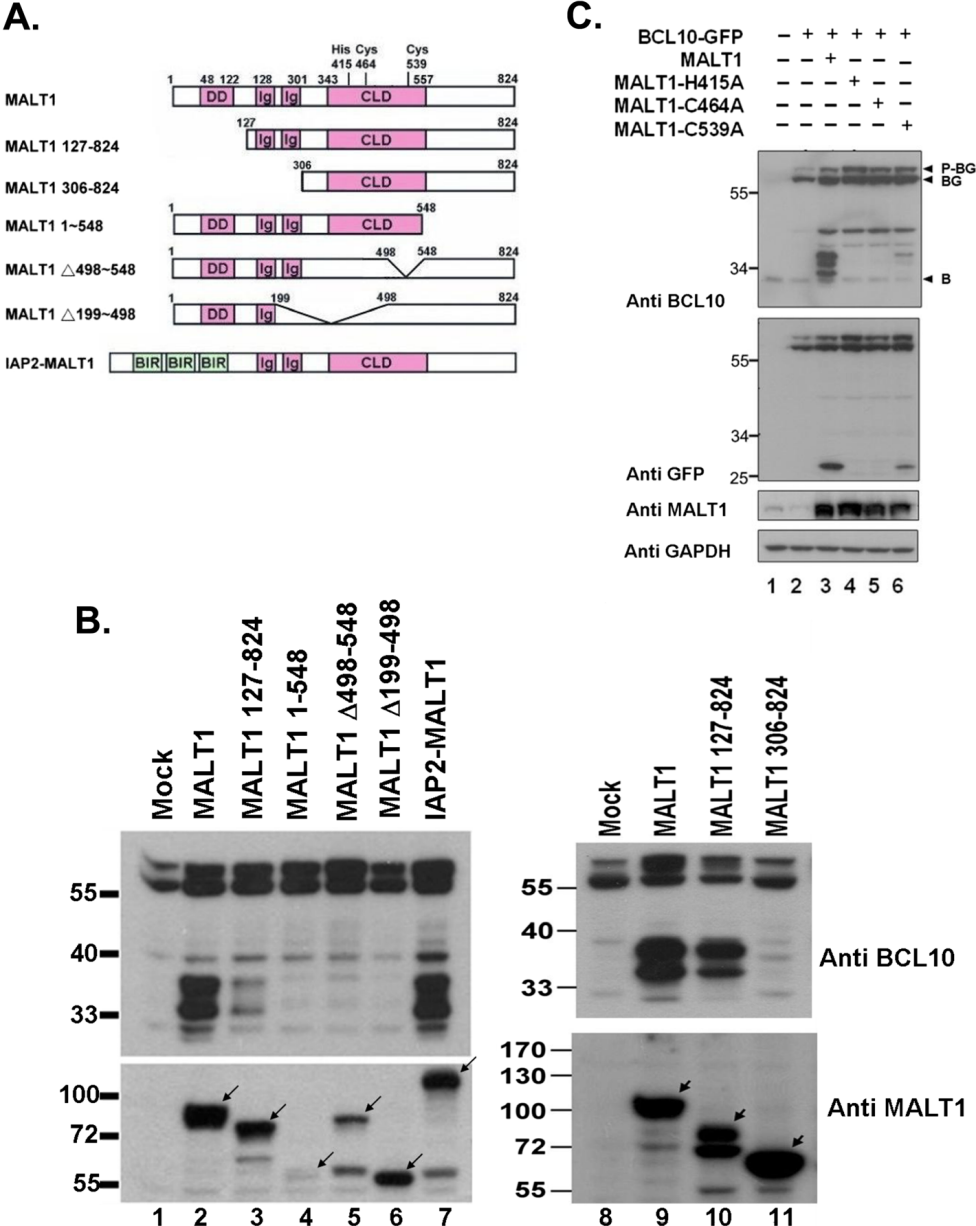
**Mapping domains on MALT1 required for inducing the cleavage of BCL10GFP**. (**A**) Schematic diagram of the deduced protein domains in human MALT1 and deletion mutants. The numbers indicate the start and the end of the domains. The predicted catalytic residues (His 415, Cys464) of the caspase-like domain were marked. (**B**) (**C**) Western blot analysis of lysates from cells transfected with various constructs as indicated using anti-BCL10, anti-MALT1, anti-GFP antibodies. Arrow head indicated the expected expression product of each MALT1 expression vector. Mouse anti-MALT1 antibody (sc-46677, Santa Cruz) was raised against amino acids 525-824 of MALT1. The poor signal in lanes 4 and 5 (panel **B**) was due to the inferior recognition by this anti-MALT1 antibody. B: BCL10; BG: BCL10GFP; P-BG: Phosphorylated BCL10GFP.

Several deletion mutants (Figure 2A) of MALT1 were generated to map the domain(s) critical for BCL10GFP cleavage. While N-terminal DD domain (MALT1 127-824, Figure 2B, lanes 3, 10) was not essential for MALT1-mediated proteolytic event, deletion of the DD and two Ig-like domains (MALT1 306-824) abolished the effect (Figure 2B, lane 11). The proteolytic activity was dependent on an intact caspase-like-domain, as deletion mutants (MALT1 1-548, MALT1 Δ498-548, MALT1 Δ199-498) showed no significant activity (Figure 2B, lanes 4, 5, 6). The IAP2-MALT1 fusion protein, containing the two Ig-like and caspase-like-domains of MALT1, retained the ability of inducing BCL10GFP cleavage (Figure 2B, lane 7). Catalytic diads (His415 and Cys464) of MALT1 paracaspase were identified as critical residues engaging the proteolytic reaction (Figure 2C, lanes 4 and 5) as previously reported [11,12].

### MALT1-induced proteolytic cleavage of BCL10GFP was dependent upon the integrity of CARD domain and MALT1 interacting domain on BCL10

**Figure 3:**
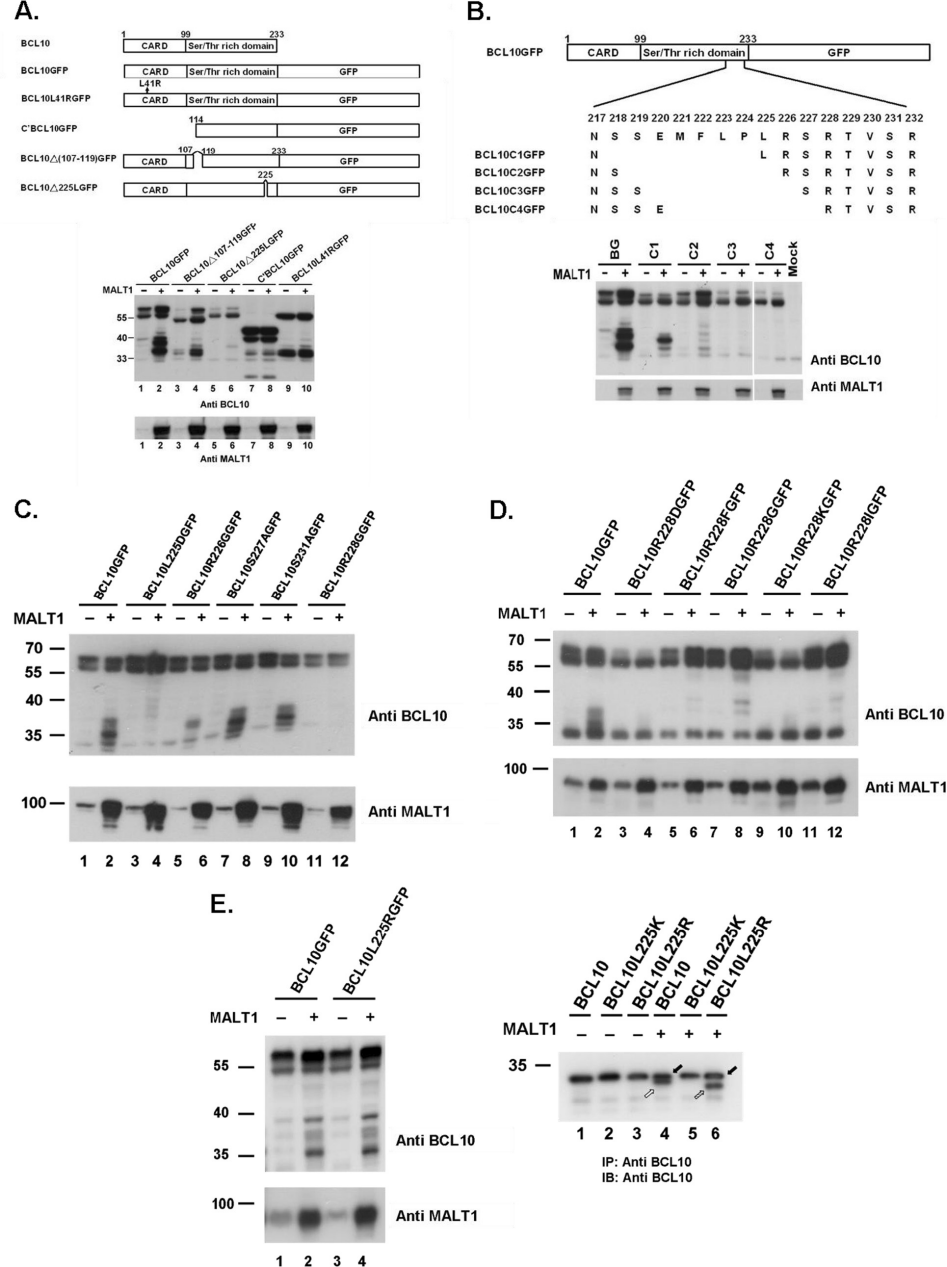
**Identification of the determinants on BCL10GFP essential for MALT1-induced cleavage.** (**A**) (**B**) (**C**) (**D**) Western blot analysis of lysates from cells transfected with various constructs indicated in the absence (-) or the presence (+) of MALT1 using anti-BCL10, anti-MALT1 antibodies. (**E**) Left: Western blot analysis of lysates from HEK293T cells transfected with BCL10GFP or BCL10L225RGFP in the absence (-) or the presence (+) of MALT1. Right: Lysates of cells transfected with BCL10, BCL10L225K or BCL10L225R in the absence (-) or the presence (+) of MALT1 were treated with alkaline phosphatase and analyzed by western blot analysis. filled arrow: full length BCL10; unfilled arrow: truncated BCL10; half-filled arrow: cryptically processed BCL10.

Truncation of the N terminus CARD domain (C’BCL10GFP) or a single mutation (BCL10L41RGFP) in the CARD domain of BCL10 abolished its phosphorylation (Additional file 1: Figure S1). In addition to the expected size of expression, several truncated forms were consistently observed in lysates of cells with expression of these two mutants alone (Figure 3A, lanes 7, 9). The identity of these truncated products remained to be determined. The expression of MALT1 did not alter the expression pattern of these mutants (Figure 3A, lanes 7, 8, 9, 10). These findings suggested that CARD domain was required for phosphorylation and MALT1-induced proteolytic cleavage on BCL10GFP. It has been shown that the amino acid residues 106-120 of BCL10 interact with MALT1 [2,10]. Deletion of aa 107-119 (BCL10Δ107-119GFP) diminished its ability of being processed by a MALT1-induced event (Figure 3A. lanes 3, 4), indicating that the interaction of BCL10 with MALT1 was critical for the proteolytic reaction.

### A single amino acid deletion or mutation at Leu225 on BCL10 greatly affected its ability of being cleaved by MALT1

Serial deletion mutants (Figure 3B) or mutants with point-mutation were generated to investigate the contribution of amino acid residues near the P1 position -- Arg228. MALT1-induced truncated forms could be observed in the lysates of cells with BCL10C1GFP expression (Figure 3B). BCL10C2GFP, BCL10C3GFP, and BCL10C4GFP, retaining the invariant Arg228 in the P1 position, were not good substrates for MALT1 (Figure 3B). In fact, a single amino acid deletion at Leu225 of BCL10 (BCL10Δ225LGFP) diminished greatly the MALT1-induced proteolytic event (Figure 3A, lanes 5, 6). A mutation at Arg226 to Glycine (BCL10R226GGFP), a mutation at Ser227 to Ala (BCL10S227AGFP), and a mutation at Ser231 to Ala (BCL10S231GGFP) had no effect on their abilities of being processed by MALT1 (Figure 3C, lanes 5-10). Mutants with mutation at the invariant P1 Arg228 including BCL10R228DGFP, BCL10R228FGFP, BCL10R228GGFP, BCL10R228KGFP, and BCL10R228I GFP (Figure 3D), lost their abilities of being processed. For P4 Leu225, mutants such as BCL10L225AGFP, BCL10L225GGFP, BCL10L225DGFP, BCL10L225EGFP, BCL10L225QGFP, BCL10L225KGFP, and BCL10L225TGFP were not processed by MALT1 (data not shown). However, BCL10L225RGFP was proteolytically processed by MALT1 as BCL10GFP (Figure 3E). Alkaline phosphatase treatment of lysates from cells transfected with BCL10L225R and MALT1 revealed the appearance of a truncated BCL10 with faster migrating mobility than MALT1-processed BCL10 (Figure 3E, right panel, lanes 4 and 6).

**Figure 4:**
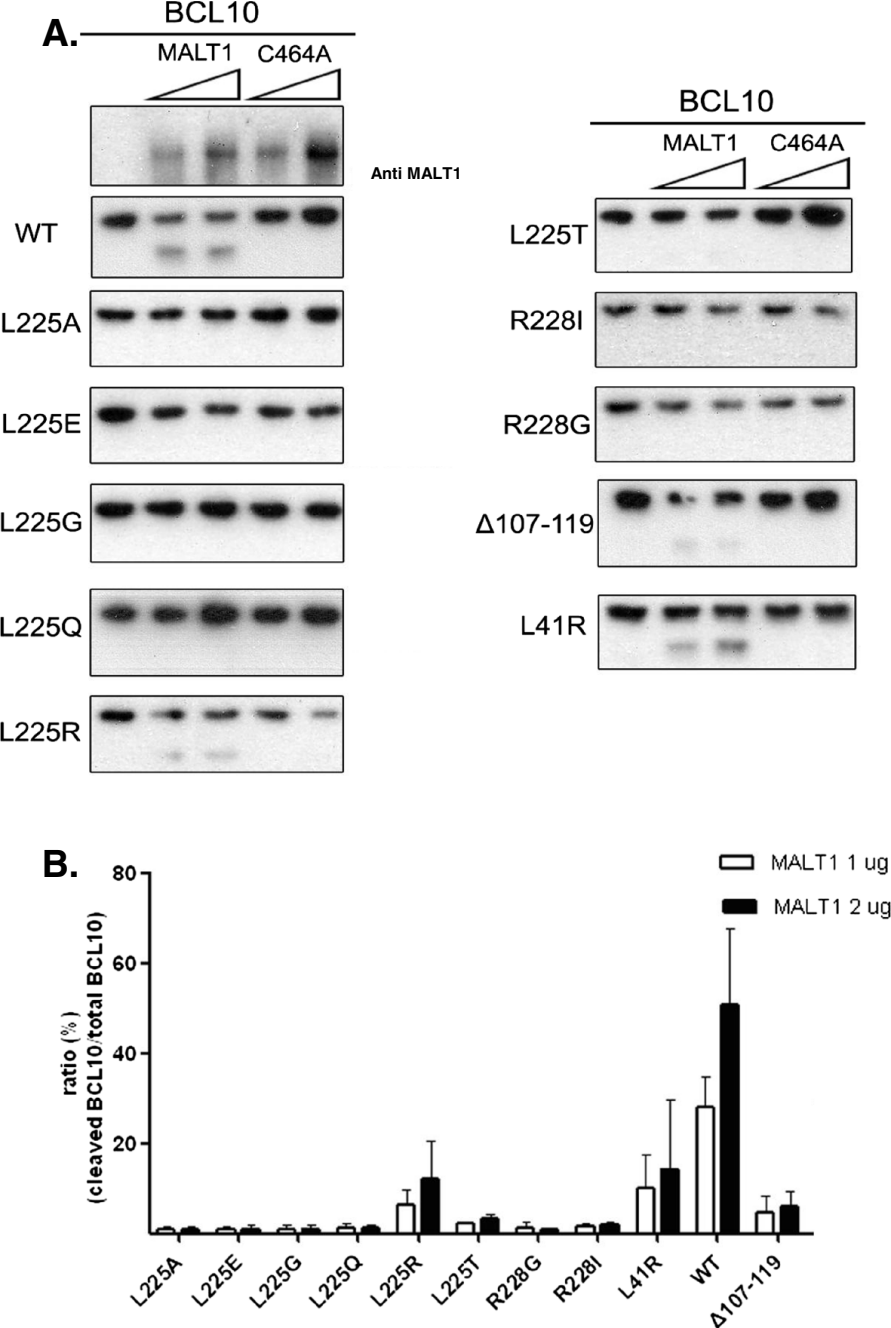
***In vitro *cleavage assay of BCL10 and mutants by MALT1.** (**A**) His-tagged BCL10 and BCL10 mutants were expressed in *E. coli*, purified and incubated with increasing concentrations of His-tagged MALT1 or MALT1C464A mutant respectively. Samples were resolved by SDS/PAGE, followed by Western blot analysis using anti-MALT1 (top left) or anti-BCL10 antibody. Data are representative of three separate experiments. (**B**) Quantitative analysis of (**A**). The percentage of BCL10 processed was defined as the signal of cleaved product divided by the sum of signal of full-length protein and the cleaved product.

*In vitro* cleavage assays using purified BCL10 and MALT1 in the presence of kosmotropic salts were also performed to verify what were observed *in vivo*. As shown in Figure 4, albeit at lower efficiency, BCL10L225R was processed by MALT1. Different from what was shown *in vivo* (Figure 3A), BCL10L41R and BCL10Δ107-119 could be processed by MALT1 *in vitro* in the presence of kosmotropic salts (Figure 4).

## Discussion

BCL10, a binding partner of MALT1, is an identified MALT1 substrate [11]. BCL10 is a 233 amino acids polypeptide. Cleavage of BCL10 by MALT1 occurs at the C-terminal end of Arg228. Overexpression of BCL10 induces its hyperphosphorylation. The presence of multiple hyperphosphorylated forms and a mere 5 amino acids difference between the substrate and the proteolytic product made it difficult to tell whether the cleavage event took place by using a simple western blot analysis. BCL10 cleavage was once reported to be monitored with a neo-epitope antibody that recognizes the new C-terminus of BCL10 [18]. In this study, we offered a way of monitoring cleavage of BCL10 by using BCL10GFP as substrate for MALT1. As shown in Figure 1, we were able to demonstrate the cleavage of BCL10GFP fusion protein as BCL10 in cells with ectopical expression of MALT1. When overexpressed, BCL10GFP formed cytoplasmic filaments as BCL10 did. Mutation or deletion of the major phosphorylation sites (Ser 134, Ser 136, Ser 138, Ser 141, Ser 144, Ser 170, and Ser 171) on BCL10 or BCL10GFP abolished the appearance of hyperphosphorylated forms as well (data not shown). All the data indicated that the fusion of GFP tag on BCL10 did not jeopardize its ability of being phosphorylated or as a substrate for MALT1. Hence, BCL10GFP can be utilized as a convenient tool for studying the determinants for efficient MALT1 cleavage in HEK293T cells.

BCL10 binds directly and constitutively to the DD and the first two Ig-like regions of MALT1 [2,3,10,19]. Consistent with others, deletion of the DD of MALT1 (MALT1 127-824) had little influence on its protease activity. Deletion of both DD and Ig-like domains (MALT1 306-824), abolishing the interaction with BCL10, greatly affected its proteolytic activity on BCL10GFP (Figure 2B). However, DD and the first two Ig-like domains of MALT1 are not required for activity *in vitro* using peptide substrates such as the BCL10-based substrate Ac-LRSR-amc [17,20]. IAP2-MALT1 fusion oncoprotein induces cleavage of NIK [15]. NIK is recruited to the IAP2 moiety of IAP2-MALT1 fusion protein and is cleaved by the MALT1 paracaspse domain [15]. Cleavage of NIK is not observed in cells expressing activated MALT1 [15]. Therefore, for cleavage of natural protein substrates as opposed to peptide substrates, interactions between protease and substrate are critical.

Based on sequence alignment with caspases, metacaspases, miscellaneous eukaryotic and bacterial cysteine proteases, His415 and Cys464 of MALT1 were identified as the possible catalytic diad [2]. Indeed, mutation of these two residues abolished its catalytic activity (Figure 2C). MALT1 1-548 failed to process BCL10GFP. In this context, an extension of catalytic active site into a region C-terminal to the cited caspase-like-domain is suggested. These results coincided with two recent studies indicating that deletion of domains C-terminal to the caspase-like domain jeopardized its stability [16,17]. In fact, the caspase-like domain and the following Ig domain are shown to appear as a single folding unit [16].

The expression of MALT1 alone is insufficient to induce proteolytic activation. The activation of MALT1 can be achieved by overexpression of BCL10 [11,12] in cells. Homotypic interactions between the CARD domain results in the oligomerization of BCL10, which in turn mediates the oligomerization and activation of MALT1. Deletion (C’BCL10GFP) or mutation (BCL10L41RGFP) of the CARD domain failed to activate MALT1. Hence, both mutants were not processed *in vivo* due to the failure of generating a functional MALT1 paracaspase. BCL10L41R was still a substrate for activated MALT1. *In vitro*, kosmotropic salts mediated the oligomerization of MALT1 and the activation of its catalytic activity, bypassing BCL10-mediated oligomerization. Hence, BCL10L41R was processed by MALT1 *in vitro* in the presence of kosmotropic salts.

Unlike the related metacaspases, which accept both Lys and Arg as a P1 residue [21], the analysis of crystal structure of MALT1 paracaspase region suggests a degree of conserved substrate specificity with Arg invariant at the P1 position [16,17]. The positively charged P1 residue Arg is coordinated by three highly conserved acidic residues in the S1 pocket. The carboxylate side chain of each of the three acidic residues accepts a pair of charge-stabilized hydrogen bonds from the guanidinium group of P1-Arg. Our results that a mutation of Arg228 to Asp, Phe, Gly, Lys, or Ile of BCL10GFP abolished its activity of being processed by MALT1 are consistent with these findings. A mutation of P2-Ser227 to Ala227 on BCL10GFP had no effect on its ability of being a substrate for MALT1, coincided with the finding that P2 position was moderately selective for amino acids with small side chains due to a restricted S2 pocket [16]. A mutation to Gly at the P3 Arg226 (BCL10R226GGFP) retained its ability of being processed by MALT1. The result was in agreement with the notion that there was small contribution of P3 residue for substrate recognition.

The shallow, hydrophobic S4 pocket of MALT1 is open to accommodate hydrophobic residues. Hence, preference for a hydrophobic residue at P4 position is suggested [16,17]. While the manuscript was in preparation, Hachmann et al. reported the optimal cleavage sequence for MALT1 using positional-scanning peptidyl substrate libraries [20]. Leucine was shown to be strongly favored in the P4 position. The observation that serial deletion mutants BCL10C2GFP, BCL10C3GFP and BCL10C4GFP, though retaining the P1-Arg228, lost their abilities of being MALT1 substrates might simply be due to the loss of a hydrophobic P4 residue. BCL10Δ225LGFP utilizing Pro224 as the cryptic P4 residue lost its ability of being processed by MALT1. A serial of BCL10GFP mutants (L225A, L225G, L225D, L225E, L225Q, L225T, L225R, L225K) with mutation at the P4-Leu225 residue were tested. All but one (BCL10L225RGFP) lost their abilities of being substrates for MALT1. The observation that BCL10L225R could be processed by MALT1 using *in vivo* and *in vitro* assay seemed to be conflicted with the notion that polar residues in the P4 position were not tolerated [20]. However, the truncated products from BCL10L225R processed by MALT1 migrated faster than those from wild type BCL10 processed by MALT1 (Figure 3E). Mutation at Phe222 to His, Ser, or Glu on BCL10L225R abolished the cleavage (data not shown). Therefore, a cryptic cleavage site at the carboxyl-end of L225R was highly suspected.

Using BCL10, the natural substrate of MALT1, as a tool, the major determinants for MALT1 cleavage are consistent with what were found *in vitro* with peptidyl substrates. Since it was not possible to screen for preferences in P1’ positions (C-terminal to the scissile bond) using peptidyl substrate, our system utilizing BCL10GFP can be a good tool to address the issue.

## Conclusions

We offered a way of monitoring the catalytic activity of MALT1 in HEK293T cells using BCL10GFP as a substrate. BCL10GFP can be utilized as a convenient tool for studying the determinants for efficient MALT1 cleavage in HEK293T cells.

## Competing interests

The authors declare that they have no competing interests.

## Authors' contributions

SYJ carried out the site directed mutagenesis and *in vivo* cleavage assay, drafted the manuscript. CCC participated in the *in vitro* cleavage assay and analysis of data. CHW participated in the acquisition and analysis of data. MRC and CHT are involved in revising the manuscript critically for important intellectual content. WHC and YHC conducted site directed mutagenesis and *in vivo* cleavage assay, participated in acquisition of data. SLD conceived of the study, and participated in its design and coordination and helped to draft the manuscript. All authors read and approved the final manuscript.

## Supplementary Material

Additional file 1 **Figure S1.** Mutation (BCL10L41RGFP) or deletion (C’BCL10GFP) of CARD on BCL10 abolished its ability of being phosphorylated and processed by MALT1. Click here for file
