# Supplementary material for: BCL10GFP fusion protein as a substrate for analysis of determinants required for Mucosa-Associated Lymphoid Tissue 1 (MALT1)-mediated cleavage
Source: J Biomed Sci. 2012 Oct 5;19(1):85. doi: 10.1186/1423-0127-19-85 (PMC3500650; doi:10.1186/1423-0127-19-85)
Supplement: Additional file 1 — Figure S1. Mutation (BCL10L41RGFP) or deletion (C’BCL10GFP) of CARD on BCL10 abolished its ability of being phosphorylated and processed by MALT1. [file 1423-0127-19-85-S1.pdf]

Supplementary information

A. BCL10 constructs:

A 1.5 kb fragment was isolated from *Eco RI / Not I*-cut reaction mixture of EST clone IMAGE:703916, blunt-ended with Klenow DNA polymerase and ligated into the *Not I* / blunt-ended pRc/CMV, generating **pRc/CMVBCL10**. Two primers: 5'- TCC ACC ATG GAG CCA CCG CAC -3', 5'- AAG CTT CTT GTC GTG AAA CAG TAC G -3' were utilized to amplify a stop-codon-deleted BCL10 DNA fragment using the EST clone IMAGE:703916 as template. The 0.7 kb fragment was cloned into the pGEM-T Easy vector and subjected to sequence analysis. The clone with correct sequence was digested with *Eco RI* and *Hind III*, generating a 0.7 kb DNA fragment. The 0.7 kb DNA fragment was ligated with *Eco RI* / *Hind III*-cut pGFPemd-basic, generating pGFPemd-basic-BCL10. A 1.5 kb DNA fragment was isolated from the *Eco RI* / *Fse I*-digested pGFPemd-basic-BCL10, blunt-ended with Klenow DNA polymerase and ligated into the *Hind III* / blunt-ended site of pRc/CMV, generating **pRc/CMVBCL10GFP**.

The point mutation in the CARD motif (L41R) was obtained by amplification on cloned full-length wild-type BCL10 cDNA using the following two pairs of primers: 5'- TCC ACC ATG GAG CCC ACC GCA C -3', 5'- GAT TTT TTT TTG CAC GCC GAT GAT CAA AAT GTC TCT CAG C -3', and 5'- ATC GGC GTG CAA AAA AAA TAC TCA GTA GAG AAG A -3', 5'- AAG CTT CTT GTC GTG AAA CAG TAC G -3'. The 0.7 kb DNA fragment was cloned into the pGEM-T Easy vector and sequence-confirmed, generating pGEM-T BCL10L41R. A 05 kb DNA fragment was isolated from *Eco RI* / *Xba I*-cut pGEM-T BCL10L41R and ligated with the 4.1 kb DNA fragment isolated from *Eco RI* / *Xba I*-cut pGFPemd-basic-Bcl10, generating pGFPemd-basic-BCL10L41R. A 1.5 kb DNA fragment was isolated from the *Eco RI* / *Bam HI*-digested pGFPemd-basic-Bcl10L41R, blunt-ended with Klenow DNA polymerase and ligated into the *Hind III* / blunt-ended site of pRc/CMV, generating **pRc/CMVBCL10L41RGFP**. Primers: 5'- ATG AAA GGA CTA AAA TGT AGC -3', 5'- AAG CTT CTT GTC GTG AAA CAG TAC G -3' were utilized to amplify a DNA fragment with deletion of 113 amino acids in the N' terminus of BCL10, generating **pRc/CMVC'BCL10GFP.** The rest of the BCL10 mutants were generated by utilization of a PCR-based site directed mutagenesis method. The primers utilized were as the followings: 5'- ACG TGA TCT TAA GTT TGC ACA AGT TCC TTC -3' for **pRc/CMVBCL10C1GFP**; 5'- AGT ACG TGA TCT AGA GTT TGC ACA AGT TCC -3' for **pRc/CMVBCL10C2GFP**; 5'- AAC AGT ACG TGA ACT AGA GTT TGC ACA AGT -3' for **pRc/CMVBCL10C3GFP**; 5'-

TGA AAC AGT ACG CTC ACT AGA GTT TGC ACA -3' for **pRc/CMVBCL10C4GFP**; 5'- GAA GTG CTG AAA CTT AGC AGT TGT GAA CT TTT -3' for **pRc/CMVBCL10Δ(107-119)GFP**; 5'- GAG ATG TTT CTT CCC AGA TCA CGT ACT GTT TCA -3' for **pRc/CMVBCL10Δ225LGFP;**5'-AGA TCA CGT ACT GTT GCC CGA CAA GAA GCT TGG-3' for **pRc/CMVBCL10S231A GFP** ; 5'- TTT CTT CCC TTA AGA GCC CGT ACT GTT TCA CGA-3' for **pRc/CMVBCL10S227AGFP**; degenerate primer 5'-TCT AGT GAG ATG TTT CTT CCC VVA AGA TCA CGT ACT GTT TCA CG-3' for **BCL10 L225 mutants**; degenerate primer 5'- GAG ATG TTT CTT CCC TTA AGA TCA DHT ACT GTT TCA CGA CAA GAA GCT AA-3' for **BCL10R228 mutants**.
A 0.75 kb DNA fragment was isolated from *Hind III* cut *Nco I* - Klenow treated pGEM-T BCL10 and ligated with the 5.4 kb DNA fragment isolated from *Hind III* cut *Nhe I* (Klenow treated) pET21a, generating **pET21aBCL10-His**. The **pET21aBCL10-His mutants** were generated by utilization of a PCR-based site directed mutagenesis method using primers as described above. .

B. MALT1 mutants:

Two pairs of primers: 5'-ATG AAC TTG TCG ACG ATA -3', 5'- TTA CAG TAA TCT TTA TTC TTG ATT CTT TTT CCT CAG -3' and 5'- CTG AGG AAA AAG CAA GAA TAA AGA TTA CTG TAA -3', 5'- TTGGTT CAA CAC AGA TTT -3' were utilized to amplify the fusion junction of IAP2-MALT1 gene. The DNA fragments were cloned into pGEM-T Easy vector and subjected to sequence analysis. A 0.4 kb fragment was isolated from the S*pe I*-digested mixture of the right clone and ligated with S*pe I*-cut pCMV6/XL5/IAP2. Subsequently, the clone was digested with *EcoR I*, *Not I* and ligated with a 2.2 kb DNA fragment isolated from *EcoR I/Not I* digested pCMV6/XL5/MALT1, generating **pCMV6/XL5/IAP2-MALT1**. Primers 5'- ATG AAG ATT ACT GTA AAC CCA -3' and 5'- GAT TCC ATT TGC CAA TCC AG -3' were utilized to amplify a DNA fragment covering aa127-aa510 of MALT1 gene. The DNA fragments were cloned into pGEM-T Easy vector and subjected to sequence analysis, generating pGEM-T Easy MALT1ΔN. A 220 bp DNA fragment was isolated from *EcoR I*-digested pGEM-T Easy MALT1ΔN and ligated with the 6.7 kb DNA fragment isolated from *EcoR I*-digested pCMV6/XL5/ MALT1, generating **pCMV6/XL5/MALT1 127-824**. Primers 5'- ATG ATC ATC ATA GGA AGA ACA GAT-3' and 5'- GAT TCC ATT TGC CAA TCC AG -3' were utilized to amplify a DNA fragment covering aa306-aa510 of MALT1 gene. The DNA fragments were cloned into pGEM-T Easy vector and subjected to sequence analysis, generating pGEM-T Easy MALT1Δ2Ig. A 0.6 kb DNA fragment was isolated from *EcoR I/Hind III* -digested pGEM-T Easy MALT1Δ2Ig and ligated with the 5

kb DNA fragment isolated from *EcoR I/Hind III*-digested pCMV6/XL5/ MALT1, generating **pCMV6/XL5/MALT1 306-824**. For **pCMV6/XL5/ MALT1 1-548**, pCMV6/XL5/ MALT1 was fist digested with *Xba I*, blunt-ended with Klenow DNA polymerase, and self ligated. A 8.0 kb DNA fragment was isolated from *Hind III*/*Xba I* partially-digested pCMV6/XL5/ MALT1, blunt-ended with Klenow DNA polymerase, and self ligated, generating **pCMV6/XL5/ MALT1 Δ498-548**. A 7.3 kb DNA fragment was isolated from *EcoR I* partially-digested /*Hind III* completely-digested pCMV6/XL5/ MALT1, blunt-ended with Klenow DNA polymerase, and self ligated, generating **pCMV6/XL5/ MALT1 Δ199-498**. Mutants **pCMV6/XL5/ MALT1 H415A, pCMV6/XL5/ MALT1 C464A, and pCMV6/XL5/ MALT1 C539A** were generated by utilization of a PCR-based site directed mutagenesis method. The primers utilized were as the followings: 5'- CCC AAT AAT ATA ATA CGT CCT GCT GGT TAT GAA AAT TTT GGG; 5'- TTA CAC AAG AAT AAC CTA TAC GCT TCC TTT TCT TTA CTG ATG ; 5'- CGT CTT CTA TAC CCA TTC GCT GTG GAA TGG TTT CCG TTT GTC.
A 2.4 kb DNA fragment was isolated from *Sma I* and *Ale I* cut pCMV6/XL5/MALT1 or pCMV6/XL5/MALT1C464A and ligated with 5.4 kb *Hind III*-cut-klenow-treated pET21a, generating **pET21a-MALT1-His** and **pET21a-MALT1C464A-His** respectively.

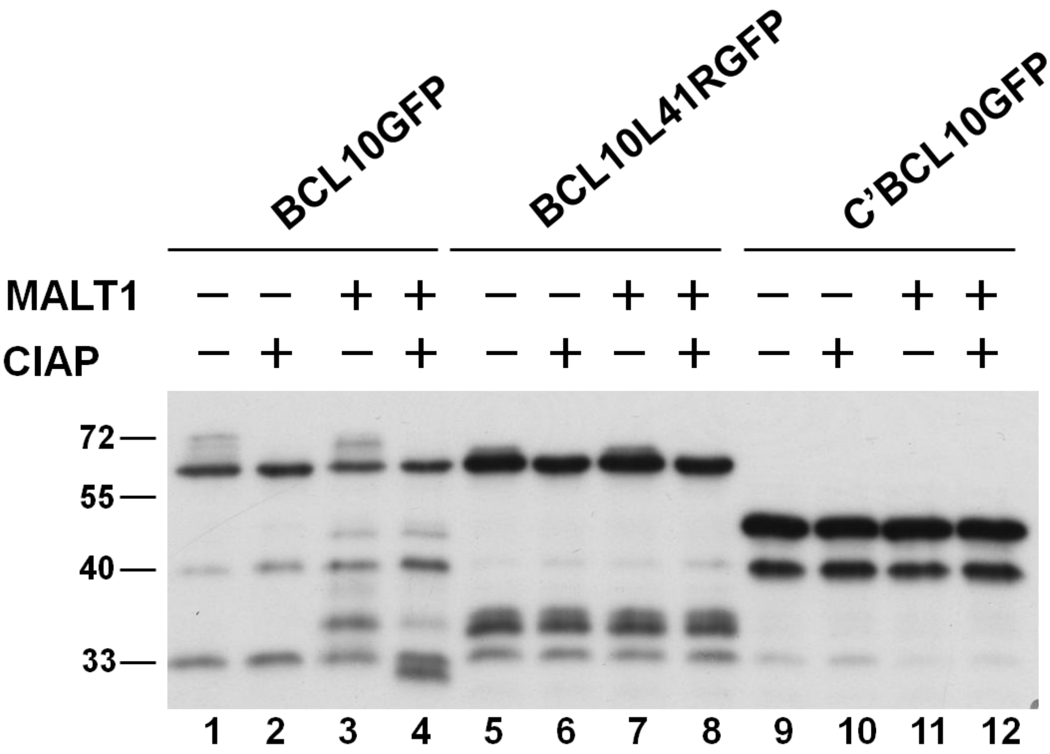

Fig. S1 Mutation (BCL10L41RGFP) or deletion (C'BCL10GFP) of CARD on BCL10 abolished its ability of being phosphorylated and processed by MALT1

Lysates of HEK293T cells transfected with BCL10GFP, BCL10L41RGFP or C'BCL10GFP in the absence (−) or the presence (+) of MALT1 were immunoprecipitated using anti-BCL10 antibody, treated with or without calf intestine alkaline phosphatase (CIAP), and analyzed by Western blotting with anti-BCL10 antibody. The migration patterns of BCL10L41RGFP and C'BCL10GFP in SDS/polyacrylamide gel were not affected by MALT1 or alkaline phosphatase treatment.
